# Hsp90 inhibitor 17-AAG reduces skin inflammation and inhibits NF-κB/NLRP3 inflammasome activation in DNCB-induced mice and TNF-α/IFN-γ-induced keratinocytes

**DOI:** 10.1515/biol-2025-1377

**Published:** 2026-10-19

**Authors:** Yuan-Yuan Chen, Xiao-Xiao Lin

**Affiliations:** Department of Dermatology, Cangnan People’s Hospital, Wenzhou, China; Department of Dermatology, Third People’s Hospital of Cangnan County, Wenzhou, China

**Keywords:** atopic dermatitis, heat shock protein 90, 17-AAG, inflammation, NF-κB, NLRP3 inflammasome

## Abstract

Atopic dermatitis (AD) represents the most common inflammatory skin condition with a high burden of disease persistence and relapse. In this study, we aimed to investigate the role of 17-AAG, a Hsp90 inhibitor, in inhibiting skin inflammation in AD and its effect on nuclear factor kappa-B (NF-κB)/NOD-like receptor thermal protein domain associated protein 3 (NLRP3) inflammasome pathway. Topical application of 17-AAG was found to significantly improve inflammatory skin lesions, reduce epidermal thickness and mast cell infiltration, and decrease levels of proinflammatory cytokines in mice with AD-like skin lesions induced by using 2,4-dinitrochlorobenzene (1 % and 0.5 % DNCB). A reduced extent of p65 phosphorylation with declined protein expressions of NLRP3 and caspase-1 in the dorsal skin lesions of 17-AAG-treated AD mice compared to vehicle-treated AD mice. In HaCaT cells inflamed by tumor necrosis factor α (TNF-α)/interferon γ (IFN-γ), 17-AAG treatment significantly reduced the secretion of proinflammatory cytokines and chemokines, reduce the phosphorylation of p65, and decreased the protein expressions of NLRP3 and caspase-1. These results prove Hsp90 inhibitor 17-AAG could reduce skin inflammation and inhibit NF-κB/NLRP3 inflammasome activation in DNCB-induced mice and TNF-α/IFN-γ-induced human keratinocytes.

## 1 Introduction

Atopic dermatitis (AD) is a chronic, relapsing inflammatory skin disease that may affect individuals from infancy to adulthood, leading to significant health and economic burden worldwide [1]. The global prevalence of AD continues to increase, with an estimated 148 million cases in 2050 [2]. AD is characterized by red eczema-like lesions, intense itch, and skin pain, all which may cause sleep disturbance, impaired mental health, and reduced quality of life [3]. AD develops primarily as a result of epidermal barrier dysfunction, immune system dysregulation, and altered skin microbiome [4], 5]. Although significant therapeutic advancements are made in AD, such as topical Janus kinase (JAK) inhibitors and biologics targeting IL-13, long-term disease control and maintenance is still challenging, particularly for those with moderate-to-severe disease [6].

Heat shock proteins (HSPs) are a highly conserved family of molecular chaperones that assist in the folding/refolding, maturation, assembly, and stabilization of over 500 client proteins to maintain cellular homeostasis [7]. Among them, heat shock protein 90 (Hsp90) is one of the most abundantly expressed and evolutionarily conserved molecular chaperone, and it is upregulated under both physiological and stress conditions [8]. The past decades have deepened fundamental understanding of Hsp90’s role in human diseases including cancer, inflammatory conditions, and neurodegenerative disorders [9]. Hsp90 is composed of three structural domains: amino (N)-terminal, middle and carboxy (C)-terminal domains among which the N-terminal domain with a nucleotide-binding pocket serves as the primary binding site for most small-molecule Hsp90 inhibitors, such as 17-AAG [10]. Topical application of 17-AAG has been demonstrated to significantly inhibit nuclear factor kappa-B (NF-κB) activation and upregulate anti-inflammatory Hsp70 expression in the skin, thus ameliorating the disease severity in a mouse model of epidermolysis bullosa acquisita [11]. Hsp90 inhibition can disrupt the Janus kinase-signal transducer and activator of transcription (JAK-STAT) pathway and suppress the activation of NF-κB, making Hsp90 inhibitors particularly attractive for treating skin inflammatory conditions including AD [12]. Treatment with 17-AAG could effectively attenuate 1,2-dichloroethane-induced neurotoxicity in mice by reducing the NOD-like receptor thermal protein domain associated protein 3 (NLRP3) inflammasome activation, suggesting inhibition of NLRP3 inflammasome may be a mechanism behind this therapy [13]. In this study, we used 2,4-dinitrochlorobenzene (DNCB) to induce mice and TNF-α/IFN-γ to treat human keratinocytes HaCaT for the purpose to prove the hypothesis that Hsp90 inhibitor 17-AAG may reduce skin inflammation and suppress the NF-κB/NLRP3 inflammasome activation in AD mice and inflamed HaCaT.

## 2 Materials and methods

### 2.1 AD-like animal model

A total of 18 female BALB/c mice (6–8 weeks old; XUNZHENG SHENG WU, Hunan, China) were housed in stable housing conditions (temperature: 22 ± 2 °C; humidity: 55 ± 10 %; 12-h light/dark cycles) with free access to standard rodent diet and water. The mice were acclimatized for at least 1 week and randomly assigned into normal group, AD group, and 17-AAG group, with six mice per group. AD-like skin inflammation was induced in the mice in the AD group and 17-AAG group. The concentrations of DNCB and modeling procedures were in accordance with a previously reported literature [14]. Briefly, after the dorsal region was shaved, mice were sensitized by using 200 µl of 1 % DNCB (Merck, Darmstadt, Germany) to paint their depilated dorsal skin on day 1 and day 4. DNCB was dissolved in a mixture of acetone and olive oil (3:1, vol/vol). After a latency period of 3 days (on days 5–7), the sensitized area of mice was challenged with 200 µL of 0.5 % DNCB from day 8 onward. The DNCB exposure challenge was applied for 3 weeks with 3 times per week. Two hours post DNCB application on day 10, the mice started to receive topical application of 200 µl 17-AAG (0.1 µM; MedChemExpress, Monmouth Junction, NJ, USA) or a mixture of dimethyl sulfoxide (DMSO) and acetone (1:40, vol/vol) at the same area daily until day 28 [15]. The mice in the normal group were treated with a mixture of acetone and olive oil and a mixture of DMSO and acetone. The mice were euthanatized by sodium pentobarbital anesthesia on day 29. The experimental schedule is outlined in Figure 1A.

**Figure 1:**
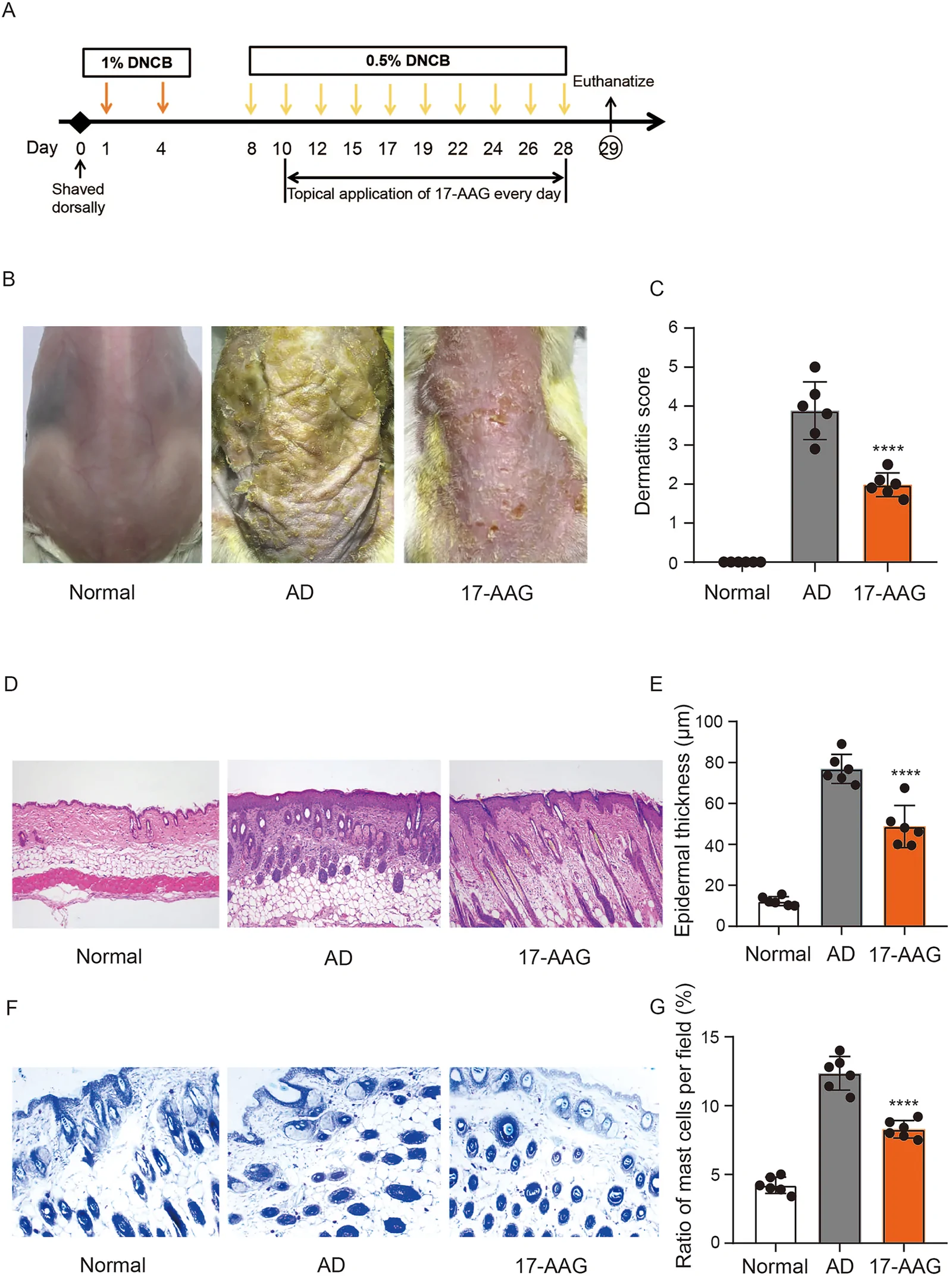
Topical application of 17-AAG improves AD-like lesions in a mouse model. A, The timeline of the DNCB-induced AD model and topical application of 17-AAG in BALB/c mice. B, Representative images of dorsal skin tissues of mice in the normal group, AD group, and 17-AAG group. C, The dermatitis scores of mice in the normal group, AD group, and 17-AAG group. D, Representative images (100×) of HE staining of dorsal skin tissues of mice. E, Quantitative analysis of epidermal thickness. F, Representative images (100×) of toluidine blue staining of dorsal skin tissues of mice. G, Quantitative analysis of mast cell infiltration in dorsal skin tissues of mice. *n* = 6 per group. ****P < 0.0001 compared to the AD group by the one-way ANOVA plus Tukey’s post-hoc test.

**Ethical approval:** The research related to animal use has been complied with all the relevant national regulations and institutional policies for the care and use of animals, and has been approved by the Institutional Animal Care and Use Committee (IACUC) at XUNZHENG SHENG WU (Approval No. 2025-XZ185).

### 2.2 Evaluation of AD severity

The severity of AD-like symptoms was evaluated by dermatitis score on day 29 based on a Scoring Atopic Dermatitis (SCORAD) system which involves four disease specific symptoms: erythema and redness, edema, excoriation, and scaling/dryness [16]. Each symptom was rated on a four-point scale (0: none; 1: mild < 20 %; 2: moderate 20–60 %; 3: severe > 60 %). The dermatitis score was defined as the sum of each symptom.

### 2.3 Histological examinations

Lesional skin tissue specimens were excised from different mouse groups were collected on day 29, fixed in 4 % buffered formalin, and embedded in paraffin. Paraffin-embedded tissue sections were serially cryosectioned (5 μm), deparaffinized, hydrated, and stained with hematoxylin/eosin (HE) (Beyotime, Shanghai, China) and 1 % toluidine blue (Beyotime). Epidermal thickness and mast cell infiltration were examined using a light microscope (Olympus, Tokyo, Japan) in six randomly selected areas (100× magnification) per slide.

### 2.4 Cell culture and treatment

HaCaT immortalized human keratinocytes (Chinese Academy of Sciences, Shanghai, China) were cultured in high-glucose Dulbecco’s Modified Eagle Medium (Gibco, Grand Island, NY, USA) with 10 % fetal bovine serum (Gibco), penicillin (100 units/ml), and streptomycin (100 mug/ml) in an incubator containing 5 % CO_2_ at 37 °C. After reaching 70–80 % confluency, HaCaT cells were treated with 10 ng/mL tumor necrosis factor α (TNF-α) and 10 ng/mL interferon γ (IFN-γ) (R&D Systems, Minneapolis, MN, USA) in the presence or absence of 0.1 µM 17-AAG (MedChemExpress) for 24 h, with DMSO used as a solvent control [15], 17].

### 2.5 Enzyme-linked immunosorbent assay (ELISA)

The levels of TNF-α, IFN-γ, interleukin-1β (IL-1β), interleukin-6 (IL-6), macrophage-derived chemokine (MDC), and thymus and activation-regulated chemokine (TARC) were determined in mouse skin tissues and HaCaT cells by using mouse TNF-α ELISA kit (PT512, Beyotime), mouse IFN-γ ELISA kit (PI508, Beyotime), mouse/human IL-1β ELISA kit (PI301 and PI305, Beyotime), mouse/human IL-6 ELISA kit (PI326 and PI330, Beyotime), human MDC ELISA kit (DY336, R&D Systems, Minneapolis, MN, USA), and human TARC ELISA kit (DY364, R&D Systems) following the manufacturer’s protocols.

### 2.6 Western blotting

HaCaT cells, loaded in a 96-well plate with 10^5^ cells per well, were exposed to TNF-α/IFN-γ stimulation in the presence or absence of 0.1 µM 17-AAG for 24 h. The total protein was extracted from HaCaT cells by using lysis buffer (Boster Biological Technology, Pleasanton, CA, USA), sodium dodecyl sulfate-polyacrylamide gel electrophoresis separation and polyvinylidene fluoride membrane transfer were performed. Then, the membrane was immersed into 5 % skim milk powder and incubated with specific antibodies as follows: anti-p65 (#8242, Cell Signaling Technology, Danvers, MA, USA), anti-p-p65-Ser536 (#3033, Cell Signaling Technology), anti-NLRP3 (ab263899, Abcam, Cambridge, UK), caspase-1 (PA5-99390, Invitrogen, Carlsbad, CA, USA), and anti-β-actin (sc-58673, Santa Cruz Biotechnology, Santa Cruz, CA, USA). β-actin was used as a reference gene. The immunoblot signal quantification was performed by using the ImageJ software.

### 2.7 Statistical analysis

Statistical significance was evaluated by one-way analysis of variance (ANOVA) followed by multiple comparisons (Tukey’s post-hoc test) using the GraphPad Prism version 8.0 (GraphPad Software, La Jolla, CA, USA) and denoted as *P* < 0.05.

## 3 Results

### 3.1 Topical application of 17-AAG improves AD-like lesions in a mouse model

Frist, the therapeutic effects of 17-AAG on AD were studied. Compared with vehicle-treated AD mice, topical application of 17-AAG was found to significantly improve DNCB-induced AD-like lesions in the dorsal skin of mice as evidenced by declined dermatitis scores (Figure 1B and C). To examine epidermal hyperplasia, skin tissues were stained with HE (Figure 1D). A reduction in epidermal thickness was observed in 17-AAG-treated AD mice compared to vehicle-treated AD mice (Figure 1E). To measure the degree of mast cell infiltration, skin tissues were stained with toluidine blue (Figure 1F). The numbers of mast cells were decreased in 17-AAG-treated AD mice compared with vehicle-treated AD mice (Figure 1G). These results indicate topical application of 17-AAG attenuates the disease severity of AD in mice.

### 3.2 Topical application of 17-AAG suppresses skin inflammation in a mouse model

Next, we investigated the effects of 17-AAG on the levels of TNF-α, IL-1β, IL-6, and IFN-γ in AD mice. Results of ELISA (Figure 2A–D) showed the 17-AAG-treated AD mice exhibited lower expression levels of TNF-α, IFN-γ, IL-1β, and IL-6 in the dorsal skin with AD-like lesions compared to vehicle-treated AD mice, indicating topical application of 17-AAG suppresses AD-like inflammation in mice.

**Figure 2:**
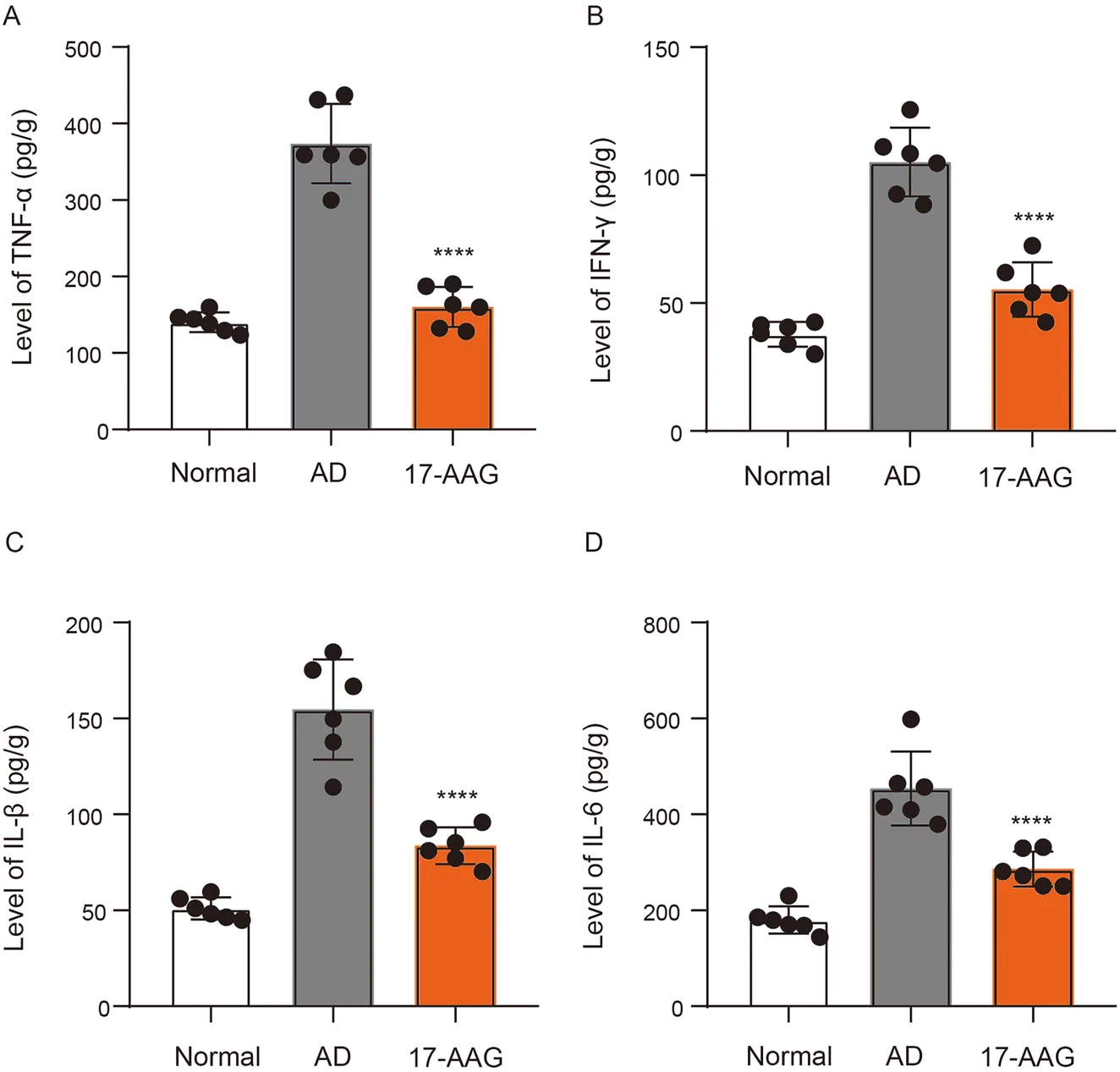
Topical application of 17-AAG suppresses AD-like inflammation in a mouse model. The expression levels of TNF-α (A), IFN-γ (B), IL-1β (C), and IL-6 (D) in the skin tissues of mice in the normal group, AD group, and 17-AAG group were determined by using ELISA kits. *n* = 6 per group. ****P < 0.0001 compared to the AD group by the one-way ANOVA plus Tukey’s post-hoc test.

### 3.3 Topical application of 17-AAG inhibits NF-κB/NLRP3 inflammasome activation in a mouse model

In addition to proinflammatory cytokines, the effects of 17-AAG on the NF-κB/NLRP3 inflammasome pathway was determined in AD mice. Results of Western blotting (Figure 3) showed a reduced extent of p65 phosphorylation with declined protein expressions of NLRP3 and caspase-1 in the dorsal skin with AD-like lesions of 17-AAG-treated AD mice compared to vehicle-treated AD mice. These results indicate topical application of 17-AAG inhibits NF-κB/NLRP3 inflammasome activation in AD mice.

**Figure 3:**
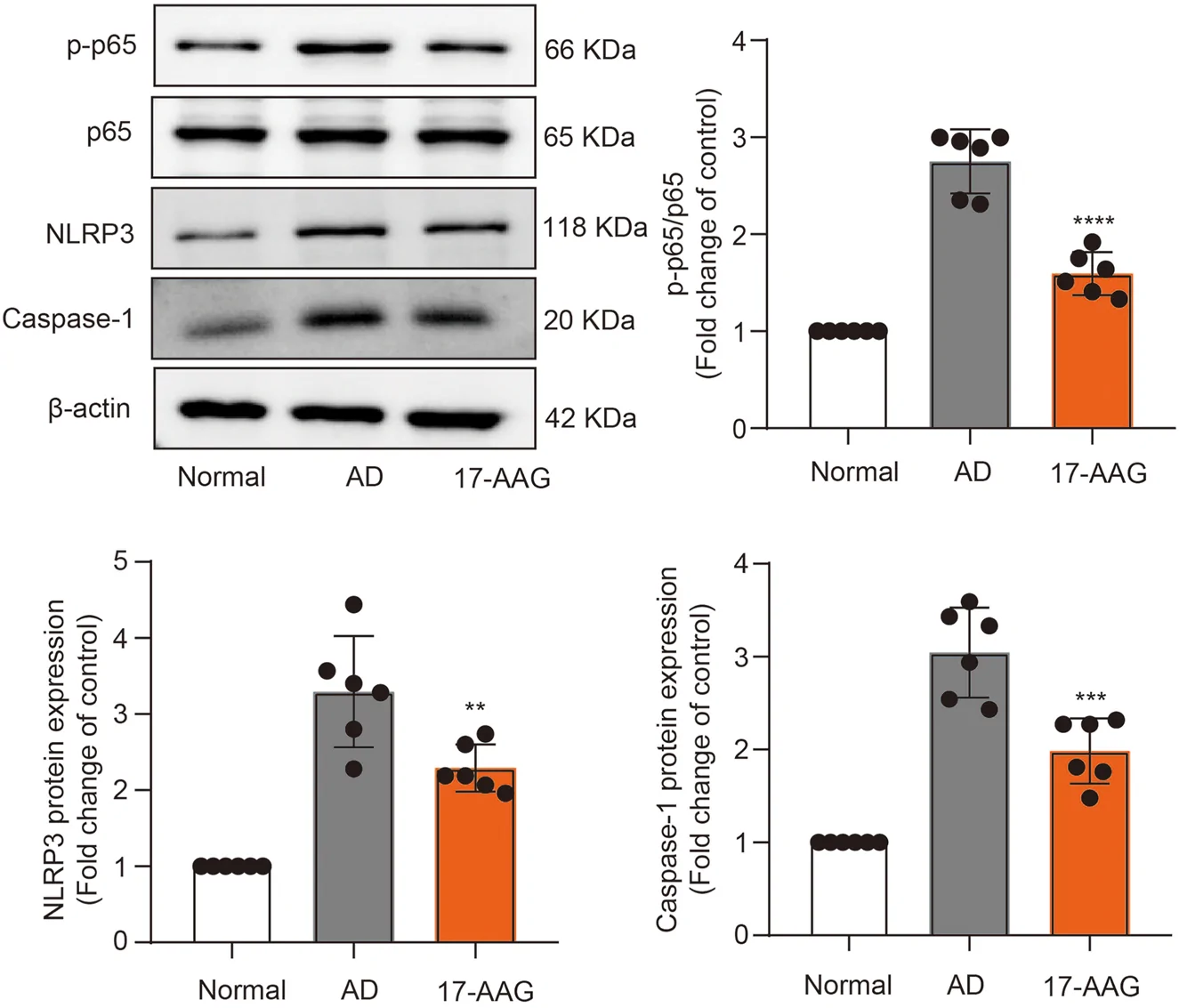
Topical application of 17-AAG inhibits NF-κB/NLRP3 inflammasome activation in a mouse model. The expressions of phosphorylated p65, NLRP3, and caspase-1 in the skin tissues of mice in the normal group, AD group, and 17-AAG group were determined by Western blotting. *n* = 6 per group. **P < 0.01, ***P < 0.001, and ****P < 0.0001 compared to the AD group by the one-way ANOVA plus Tukey’s post-hoc test.

### 3.4 Anti-inflammatory effects of 17-AAG in HaCaT cells inflamed by TNF-α/IFN-γ

To confirm the effects of 17-AAG on human keratinocytes in skin inflammation condition, we used 0.1 µM 17-AAG to treat HaCaT cells inflamed by TNF-α/IFN-γ. In addition to IL-1β and IL-6, the levels of MDC and TARC were determined in HaCaT cells as two of them are considered as important chemokines for disease severity monitoring of AD [18]. Results of ELISA (Figure 4A–D) found 17-AAG treatment significantly reduced the secretion of proinflammatory cytokines (IL-1β and IL-6) and chemokines (MDC and TARC) in TNF-α/IFN-γ-elicited HaCaT cells.

**Figure 4:**
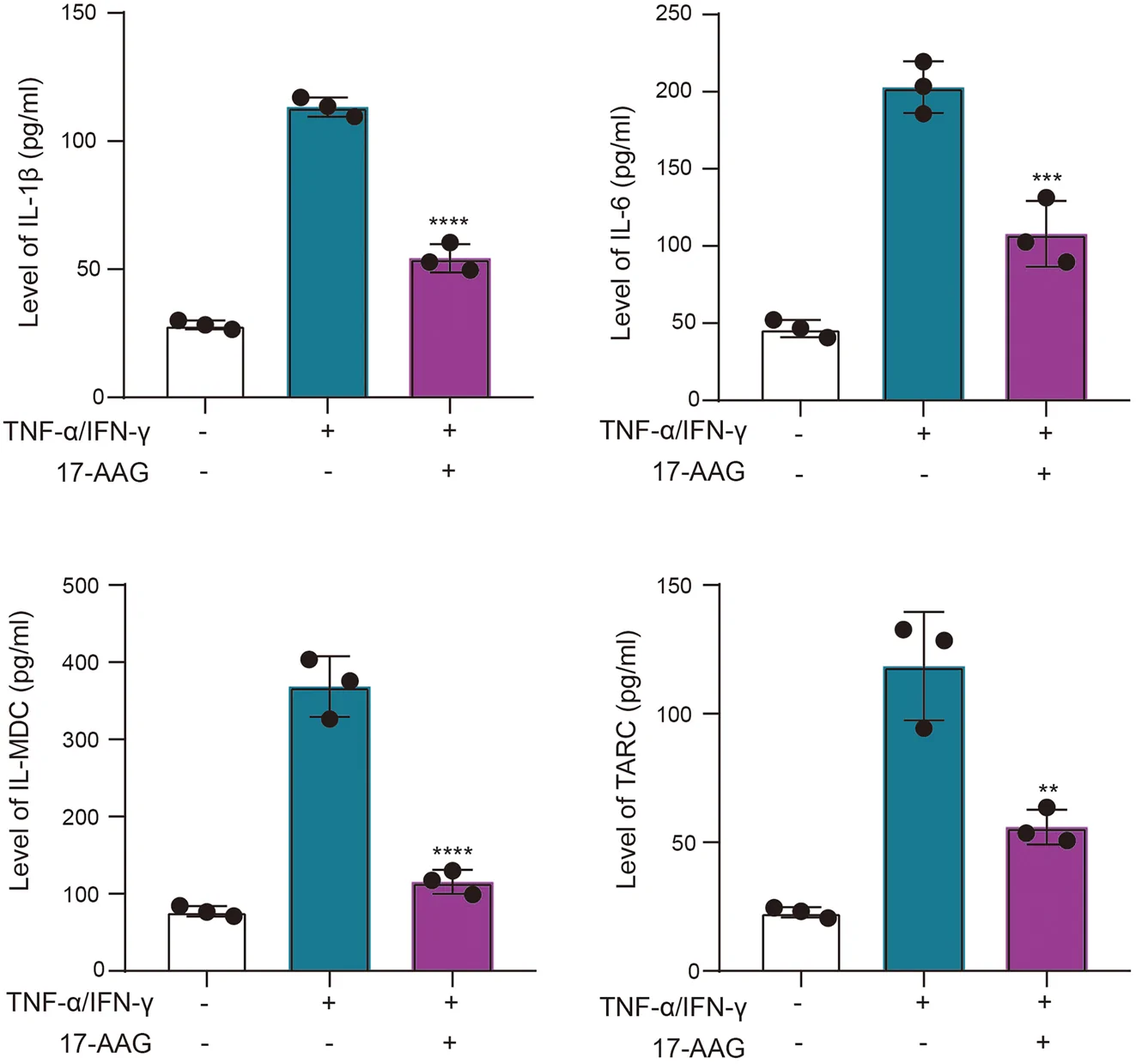
Anti-inflammatory effects of 17-AAG treatment in HaCaT cells inflamed by TNF-α/IFN-γ. HaCaT cells were treated with 10 ng/mL TNF-α/IFN-γ in the presence or absence of 0.1 µM 17-AAG for 24 h. The levels of IL-1β, IL-6, MDC, and TARC in HaCaT cells were determined by using ELISA kits. *n* = 3 per group. **P < 0.01, ***P < 0.001, and ****P < 0.0001 compared to TNF-α/IFN-γ treatment alone by the one-way ANOVA plus Tukey’s post-hoc test.

### 3.5 17-AAG inhibits NF-κB/NLRP3 inflammasome activation in HaCaT cells inflamed by TNF-α/IFN-γ

Exposure to TNF-α and IFN-γ caused the phosphorylation of p65, and upregulated the expressions of NLRP3 and caspase-1 in HaCaT cells. Compared to vehicle-treated cells, 17-AAG treatment was found to significantly reduce the phosphorylation of p65 and decreased the protein expressions of NLRP3 and caspase-1 in TNF-α/IFN-γ-stimulated HaCaT cells (Figure 5). These results indicate 17-AAG inhibits NF-κB/NLRP3 inflammasome activation in human keratinocytes inflamed by TNF-α/IFN-γ.

**Figure 5:**
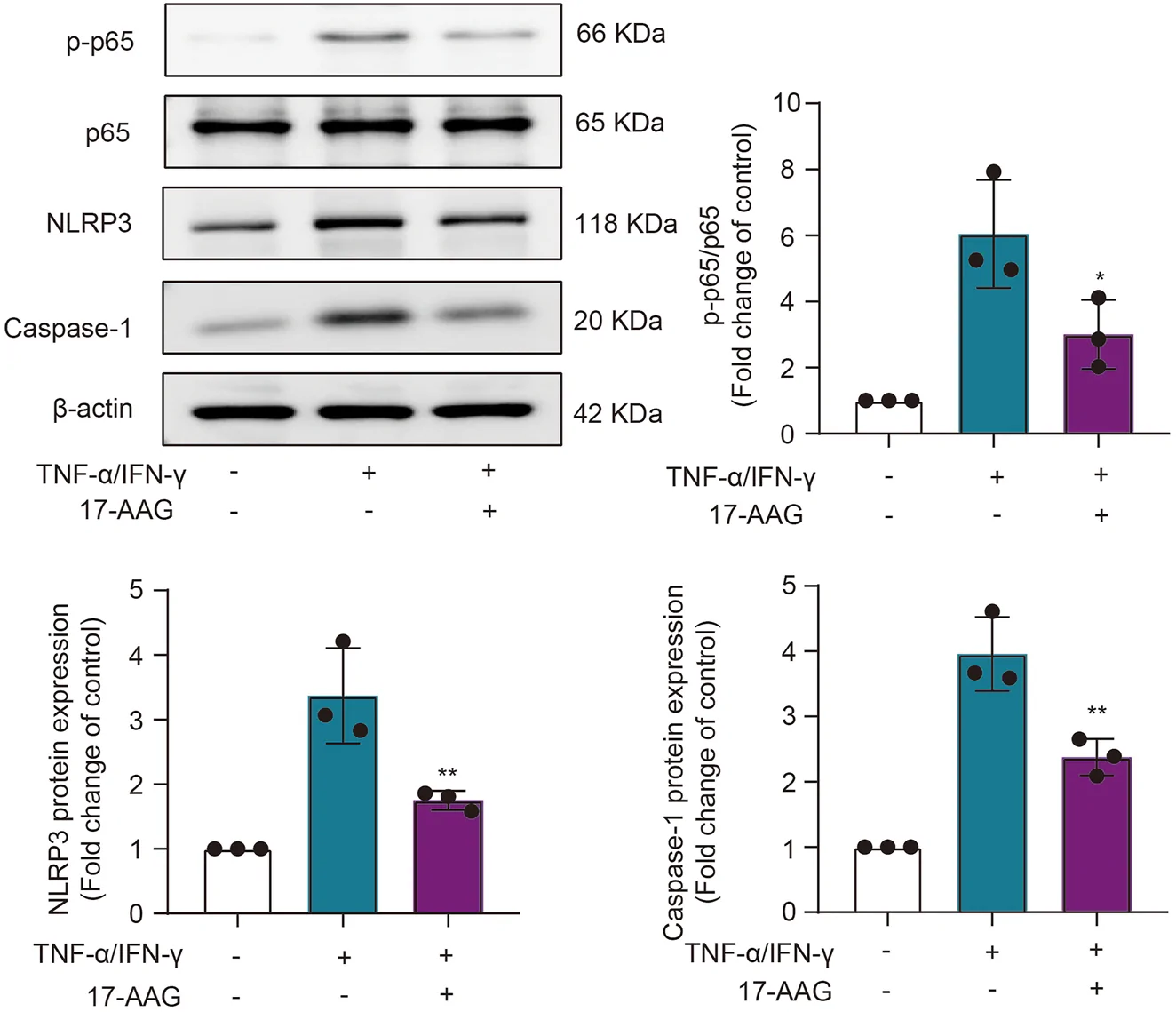
17-AAG treatment inhibits NF-κB/NLRP3 inflammasome activation in HaCaT cells inflamed by TNF-α/IFN-γ. HaCaT cells were treated with 10 ng/mL TNF-α/IFN-γ in the presence or absence of 0.1 µM 17-AAG for 24 h. The expressions of phosphorylated p65, NLRP3, and caspase-1 in HaCaT cells were determined by Western blotting. *n* = 3 per group. **P < 0.01 compared to TNF-α/IFN-γ treatment alone by the one-way ANOVA plus Tukey’s post-hoc test.

## 4 Discussion

The findings of the study demonstrated topical application of HSP90 inhibitor, 17-AAG, could reduce AD-like skin lesions in DNCB-induced mice. Furthermore, 17-AAG treatment inhibited the release of proinflammatory cytokines and chemokines in TNF-α/IFN-γ-stimulated HaCaT cells. The inhibitory effects of 17-AAG on the NF-κB/NLRP3 inflammasome activation were demonstrated in both *in vivo* and *in vitro* studies.

Hsp90 is required for stabilization of a wide range of client proteins including kinases and transcription factors, many of which are central to immune signaling cascades involved in the pathogenesis of AD [19]. As a result, Hsp90 inhibitor 17-AAG have been developed to disrupt these interactions. The first study indicating a potential role of Hsp90 in the immunopathology of AD showed AD patients exhibited significantly higher levels of serum Hsp90 and anti-Hsp90 IgE autoantibodies than healthy controls [20]. In their study, seropositivity for anti-Hsp90 IgE was observed in approximately 50 % of AD patients but it was observed in only 3 % of healthy individuals. The serum Hsp90 levels was positively correlated with disease severity of AD. Not only in contrast to health individuals, Sitko et al. [21] found 83.9 % of AD patients were Hsp90α positive, but none of the DH patients exhibited such a positivity. Sitko et al. [22] continued to investigate the therapeutic effects of Hsp90 inhibitors in preclinical animal studies of AD, demonstrating topical application of Hsp90 inhibitor STA-9090 (Ganetespib) reduced serum levels of IgE, increased filaggrin expression in lesional skin samples, and improved disease activity in AD mice. Abdallah et al. [12] used another Hsp90 inhibitor RGRN-305 to treat MC903-induced mouse model and primary human keratinocytes stimulated with TNF-α/IL-4, demonstrating topical and oral administration of RGRN-305 robustly improved clinical signs of dermatitis and reduced immune cell infiltration into the skin in mice. They also discovered RGRN-305 treatment disrupted the JAK-STAT signaling to inhibit AD-like inflammation in primary human keratinocytes. Much recently, Sitko et al. selected a different Hsp90 inhibitor 17-AAG to confirm the therapeutic effects of Hsp90 inhibition in AD. They found topical application of 17-AAG not only attenuated reduced AD-like lesion and inflammation in DNCB-induced mice but also partially restored microbial balance in AD mice, which aligned with our results. Although not in AD model, Lv et al. [23] found intraperitoneal injections of 17-AAG reduced the epidermal thickness and inflammatory cell infiltration into the skin in a mouse model of psoriasis, and Todurga Seven et al. [24] found 17-AAG reduced the itch behavior in a mouse model of pruritus, both of which were similar to our results supporting the therapeutic effects and anti-inflammatory action of 17-AAG in skin inflammatory diseases.

The activation of NF-κB/NLRP3 inflammasome pathway has been found to disrupt skin barrier integrity and cause cytokine storm in AD [25], 26]. 17-AAG was previously demonstrated to deactivate NF-κB signaling pathway and thus regulate gut microbiota [27]. Besides, 17-AAG could attenuated the pro-inflammatory activity of M1 macrophages in inflamed nucleus pulposus cells induced by M1-polarized macrophages by upregulating Hsp70 expression and inhibiting NF-κB pathway, suggesting the anti-inflammatory effects of 17-AAG in intervertebral disc degeneration [28]. Several types of inflammasomes have been identified, such as NLRP3, NLRP1, ice protease-activating factor (IPAF) and absent in melanoma 2 (AIM2), among which the most well-known inflammasome is NLRP3 which is expressed by several cells including skin keratinocytes [29]. When activated, NLRP3 works with the adaptor protein apoptosis associated speck like protein containing a CARD (ASC) and precursor caspase-1 to form an oligomer complex which is known as NLRP3 inflammasome. Augmented NLRP3 inflammasomes leads to the activation of caspase-1, which then cleaves the pro-inflammatory cytokines IL-1β and IL-18 into active forms, triggering gasdermin D (GSDMD) cleavage, leading to pyroptosis and amplifying the local inflammatory response [30]. In a previous study of subarachnoid hemorrhage, intraperitoneal administration of 17-AAG reduced the levels of NLRP3 and caspase-1, thus improving short- and long-term neurobehavioral outcomes in mice [31]. Besides, 17-AAG treatment was reported to reduce pyroptosis in THP-1 cells via inhibiting the NLRP3 inflammasome/caspase-1 signal pathway [32]. Compared with other inflammasome pathways, NLRP3 is a key bridge connecting IL-33 and neutrophilic inflammation in AD, particularly playing a central role in Th17 type inflammation [33]. Drugs targeting NLRP3 may be particularly suitable for controlling AD subtypes dominated by Th17/neutrophil inflammation [34]. 17-AAG-mediated Hsp90 inhibition was found to prevent the progression of IL-17-mediated inflammatory skin diseases, indicating 17-AAG may modulate NLRP3 rather than other inflammasomes to improve Th17 inflammation and IgG1 production [35]. As shown in our study, we proved 17-topical application of AAG deactivated the NF-κB/NLRP3 inflammasome pathway in the skin of DNCB-induced mice.

Keratinocytes, by virtue of their location, are thought to be related to the development of AD through an “outside-to-inside” mechanism, permeability barrier dysfunction, and an “inside-to-outside” mechanism, production of various proinflammatory cytokines and chemokines that increase or maintain allergic inflammation [36]. Reducing the secretion of proinflammatory cytokines and chemokines in inflamed keratinocytes may aid in developing effective therapeutic target for AD [37]. The study used a combination of TNF-α and IFN-γ to induce inflammation in HaCaT cells, with IL-1β, IL-6, MDC, and TARC levels increased in HaCaT cells inflamed by TNF-α/IFN-γ. In the study performed by Kim et al. [35], 17-AAG treatment disrupted the Act1 which was a client protein of Hsp90 and required for the IL-17 signaling in keratinocytes, thus improving the IL-17-mediated inflammatory skin diseases. As shown in our studies of human keratinocytes HaCaT cells, 17-AAG treatment reduced the secretion of proinflammatory cytokines and chemokines and deactivated the NF-κB/NLRP3 inflammasome pathway in HaCaT cells inflamed by TNF-α/IFN-γ.

The study has several limitations. One limitation is use of TNF-α/IFN-γ mixture rather than IL-4/IL-13 mixture to treat HaCaT cells and lack of proteins involved in the epidermal barrier like filaggrin, loricrin, and involucrin, which may lead to the *in vitro* data focusing on inflammation but not pathogenesis of AD. Another limitation is lack of optimal drug formulations enhancing the permeation and efficacy of topical application of 17-AAG. Further investigations should focus on permeation enhancer nanovesicles mediated topical delivery of 17-AAG in treating AD.

In conclusion, these results constitute to a finding that Hsp90 inhibitor 17-AAG could reduce inflammatory skin lesions in DNCB-induced mice and in HaCaT cells inflamed by TNF-α/IFN-γ. Our results also provide evidence revealing that Hsp90 inhibition by 17-AAG suppresses NF-κB/NLRP3 inflammasome activation during skin inflammation. The accumulating evidence of HSP90 inhibition exerting broad anti-inflammatory effects together with our findings increases the likelihood of clinical success in treating AD.
